# Lung microbiota of raccoon dogs (*Nyctereutes procyonoides*) using high-throughput sequencing

**DOI:** 10.3389/fmicb.2025.1677761

**Published:** 2025-10-20

**Authors:** Wei Li, Xin Li, Jingran Cheng, Jie Liu, Jinjun Liu, Yu Wang, Wanzhe Yuan, Erjun Ren

**Affiliations:** ^1^College of Veterinary Medicine, Hebei Agricultural University, Baoding, China; ^2^Technological Innovation Center for Fur Animal Breeding of Hebei, Shijiazhuang Academy of Agriculture and Forestry Sciences, Shijiazhuang, China

**Keywords:** raccoon dog, bacterial diversity, 2bRAD-M, viral metagenomics, pathogens

## Abstract

Pneumonia frequently causes mass mortality in raccoon dogs, resulting in significant economic loss. Additionally, raccoon dogs carry various zoonotic pathogens. This study systematically assessed pulmonary pathogens in raccoon dogs and their potential public health implications utilizing 2bRAD microbiome sequencing (2bRAD-M) and viral metagenomics. We analyzed 30 lung tissue samples for microbial composition. Sequencing revealed Pseudomonadota, Ascomycota, and Actinobacteria as dominant phyla and *Acinetobacter*, *Escherichia*, and *Klebsiella* as predominant genera. The most abundant species were *Acinetobacter baumannii*, *Escherichia coli*, and *Klebsiella pneumoniae*. In total, 158 species across 84 genera were identified, including 49 potentially zoonotic species. Viral metagenomics revealed *Peduoviridae*, *Rountreeviridae*, and *Parvoviridae* as dominant families, with *Valbvirus ValB1MD2*, *Andhravirus andhra*, and *Amdoparvovirus carnivoran3* comprising over 80% of the viral composition. These findings highlight the pathogenic complexity of raccoon dog pneumonia and its zoonotic risks, providing crucial insights for disease control and public health management.

## 1 Introduction

The raccoon dog, a basal canid species, is an omnivore with a dietary preference for animal-based feed. They are primarily fed livestock and poultry by-products such as chicken frames, duck frames, and chicken intestines. This feeding practice may not only trigger respiratory diseases in animals but also increase the risk of zoonotic pathogen transmission through cross-species transmission. China remains the world’s largest producer of farmed raccoon dogs, although production has declined from a peak of approximately 16 million pelts in 2015 to about 2.49 million by 2024 (Wang, 2025). Current Chinese raccoon dog farming predominantly relies on small-scale household farming and mixed-species operations, lacking standardized management protocols, biosafety measures, or regulated feed supply chains. These systemic deficiencies increase the risk of disease transmission and compromise product quality (Yang et al., 2021). Recently, frequent pneumonia outbreaks in raccoon dogs have caused significant economic losses to farmers. This disease primarily affects the respiratory system, presenting with coughing, sneezing, and oral/nasal hemorrhage. Contributing factors include poor breeding profitability, suboptimal environmental conditions, and antibiotic misuse. Furthermore, researchers have detected multiple cross-species transmission pathogens in raccoon dogs, including norovirus (Li et al., 2021; Li et al., 2021); avian influenza viruses (H9N2 and H5N1) (Qi et al., 2009; Qian et al., 2021); SCoV-like viruses (Guan et al., 2003); and porcine circoviruses (PCV2, PCV4) (Song et al., 2019; Wang et al., 2022), as well as bacterial pathogens such as *Escherichia, Klebsiella, Proteus, Pseudomonas,* and *Streptococcus* (Zhu et al., 2021). The transmission of these pathogens primarily originates from farmed raccoon dogs. The main routes of transmission include feeding them untreated livestock and poultry by-products or close contact with other animals in co-farming environments, leading to cross-species pathogen exchange. Some of these pathogens are zoonotic and pose substantial public health concerns.

Current studies on pulmonary microbiota rely primarily on 16S rRNA sequencing, which targets the 16S rRNA gene of microorganisms. Although it provides genus-level taxonomic resolution, it often fails to achieve precise species-level identification. Whole metagenomic sequencing (WMS) enables species-level differentiation by sequencing the entire microbial genomes, but remains cost-prohibitive and technically challenging for large-scale cohorts. Additionally, WMS typically requires substantial amounts of detectable DNA, which is difficult to obtain from low-biomass lung microbiota than from gut microbiota. To enable cost-effective, high-resolution analysis of lung microbiota, we employed 2bRAD microbiome sequencing (2bRAD-M) (Sun et al., 2022), a novel sequencing approach that uses type IIB restriction enzymes to digest microbial genomes, generating unique tags that can be mapped to species-specific 2bRAD markers. This allows accurate species-level profiling of bacteria, fungi, and archaea simultaneously, even in low-biomass samples. We analyzed the DNA virome through viral metagenomics, which involves extracting total microbial genetic material from tissues or environmental samples, followed by quality control steps (e.g., removal of rRNA, host-derived sequences, and bacterial DNA) to rapidly identify viral species composition and abundance.

Currently, there is a lack of high-throughput sequencing investigations on raccoon dog lung microbiota. In this study, we analyzed pneumonic lung tissues collected from multiple raccoon dog farms in Shijiazhuang City, Hebei Province, between 2019 and 2023 using 2bRAD-M and viral metagenomics. We aimed to compare microbial community profiles across farms, investigate the etiology of pneumonia outbreaks in these facilities, and identify potential pathogens carried by raccoon dogs, particularly major zoonotic bacterial species. This study has significant implications for the effective prevention and control of zoonotic diseases.

## 2 Materials and methods

### 2.1 Sample collection

All raccoon dogs sampled between December 2019 and September 2023 had clinical symptoms associated with pneumonia. Samples were obtained from ten different raccoon dog farms, which constitute the “groups” referenced throughout this study. Detailed information for each sample is provided in Supplementary Table S1. Disposable utensils were used during collection to avoid cross-contamination of the samples, and the samples were stored in an ultralow-temperature refrigerator at −70 °C. The experiments were approved by the Animal Welfare and Ethics Committee at Laboratory Animal Center of Hebei Agriculture University.

### 2.2 DNA extraction, library construction, and sequencing

The genomic DNA was extracted using the TIANamp Micro DNA Kit (Tiangen). To monitor potential contamination, both extraction and library preparation negative controls were implemented throughout the experimental process. The 2bRAD-M library was then prepared as previously described (Sun et al., 2022). Briefly, 1 pg–200 ng of DNA was digested with 4 U of BcgI (NEB) at 37 °C for 3 h. Adaptors were ligated to the digested fragments, and the products were amplified via PCR using Phusion High-Fidelity DNA Polymerase (NEB) with Illumina-compatible primers under the following conditions: 98 °C for 5 s, 60 °C for 20 s, and 72 °C for 10 s, followed by a final extension at 72 °C for 10 min. Amplified libraries were purified with the QIAquick PCR Purification Kit (Qiagen). Subsequently, 2bRAD-M sequencing was performed at Qingdao Ouyi Biotechnology Co., with individual sequencing for each sampleon an Illumina HiSeq X Ten platform (OE BioTech, China). Raw reads were filtered based on the BcgI recognition site. The taxonomic profiling was performed using the 2bRAD-M computational pipeline,^1^ which relies on a unique 2bRAD tag database (2b-Tag-DB) containing species-specific BcgI-derived tags. Reads with >8% unknown bases or >20% low-quality bases (Q ≤ 20) were discarded. Taxonomic assignment was performed using the 2bRAD-M pipeline with a custom tag database derived from 173,165 microbial genomes (Wang et al., 2023).

For viral metagenomics, a total of 30 lung tissue samples were randomly divided into three groups of 10 and equally pooled into three sequencing libraries (Libraries 1–3). This strategy ensured sufficient sequencing depth for detecting low-abundance viruses while managing costs. The pooled lung tissues were subjected to purification and concentration of virus-like particles (VLPs) through homogenization, filtration, and ultracentrifugation. Briefly, Tissue samples (1–10 g) were minced and homogenized in grinding tubes with steel beads using a tissue homogenizer under chilled conditions. The homogenate was centrifuged at 12,000 × g for 5 min to remove debris. The resulting supernatant was subjected to virus concentration using ultrafiltration devices (100 kDa MWCO, pre-equilibrated with PBS). Concentrated samples (300–500 μL) were collected by gentle pipetting after centrifugation at 4,000 × g. Genomic DNA and RNA were extracted using the QIAamp MinElute Virus Spin Kit according to the manufacturer’s protocol. Total RNA was then reverse transcribed into cDNA using the QuantiTect Reverse Transcription Kit (QIAGEN). Libraries generated from both DNA and cDNA using the Illumina TruSeq Nano DNA/RNA Library Prep Kit were quantified with a Qubit 4.0 Fluorometer and subjected to paired-end sequencing on an Illumina platform. Raw reads were filtered using Fastp v0.20.0 (with the following parameters: remove reads with Q-score < 20, length < 50 bp or adapter contamination) to obtain clean reads. Using the bbmap v38.51 software suite, the clean reads were aligned to the Virus-NT database to remove rRNA, host, and bacterial sequences. The resulting clean reads were then subjected to *de novo* assembly. SPAdes v3.14.1 was used to assemble the sequencing data (Bankevich et al., 2012). Depth statistics were performed using Bamsdst (v1.0.9) on the assembled scaffolds to validate the accuracy and coverage of the reads relative to the assembly results. Contigs with a length ≥500 bp were selected for depth statistics, and sliding window analysis with a window size of 200 bp was conducted and visualized. Taxonomic classification was performed on the quality-controlled sequences using Kraken2, followed by Bayesian re-estimation of species abundances with Bracken. The contigs obtained from the assembly were compared against the virus-NT database using BLAST to identify the candidate reference sequences with the closest evolutionary relationships and to determine the species classification of the aligned sequences. Whole-genome amplification and deep sequencing were performed at Shanghai Tanpu Biotechnology Co., Ltd.

### 2.3 2bRAD microbial database construction and relative abundance calculation

First, 173,165 microbial genomes (including those of bacteria, fungi, and archaea) were obtained from the NCBI RefSeq database. The built-in Perl scripts (GitHub: https://github.com/shihuang047/2bRAD-M) were used to sample restriction fragments from microbial genomes by each of 16 type 2B restriction enzymes. Finally, a specific taxonomic unit (without overlapping other species within the same taxonomic unit) was obtained as a species-specific 2bRAD marker to generate a 2bRAD microbial genome database. Quality-controlled 2bRAD tags from each sample were mapped to this database, and the relative abundance was calculated as previously described (Wang et al., 2023). To control the false-positive in the species identification, a G score was derived for each species identified within a sample as below, which is a geometric mean of the proportion of the species-specific markers that have been captured (by sequencing) and the number of all detected species-specific markers (by sequencing) of this species.


$$\text{Gscor}e_{\text{speciesi}}=\sqrt{S_{i}\times t_{i}}$$

S: Number of reads assigned to all 2bRAD markers belonging to species i within a sample.

t: Number of all 2bRAD markers of species i sequenced within a sample.

The relative abundance data derived from 2bRAD sequencing were normalized. Specifically, the relative abundance of each species was calculated using the following formula: the average read coverage of species-specific 2bRAD markers (which serves as a proxy for the number of individuals of that species in the sample) was divided by the total read coverage across all detected species (representing the total number of individuals in the sample). To ensure robust species calls and control false positives, a G-score threshold of 5 was applied.


$$\text{Relativeabundanc}e_{\text{speciesi}}=\frac{S_{i}/T_{i}}{\sum_{i=1}^{n}S_{i}/T_{i}}$$

S: The number of reads assigned to all 2bRAD markers of species i in a sample.

T: The number of all theoretical 2bRAD markers of species i.

The species diversity indices were calculated without normalization/rarefaction. Instead, species abundances were scaled up by a factor of 1e6 and rounded to integers, and the diversity indices were computed at this unified depth for comparative analysis across different samples (McMurdie and Holmes, 2014).

### 2.4 Statistical analysis

Bacterial diversity analysis was conducted using R software. The alpha diversity across groups was compared through Kruskal-Wallis test based on the Chao1 estimator (estimating species abundance and richness), Shannon index (reflecting species richness and evenness), and Simpson index (quantifying species diversity) using the “vegan” package. The “vegan” package was also used to calculate Bray-Curtis distance and binary Jaccard distance to estimate beta diversity, which was statistically compared using permutational multivariate analysis of variance (PERMANOVA). Comparisons between groups were performed using the Kruskal-Wallis H test in IBM SPSS Statistics software (Version 19). If the test results indicated a statistically significant overall difference (*p* < 0.05), post-hoc pairwise comparisons were subsequently conducted using the built-in pairwise comparison function in SPSS with appropriate adjustment for multiple comparisons. For viral homology analysis, to elucidate phylogenetic relationships, sequences belonging to different groups of related viruses were downloaded from the GenBank database. Nucleotide sequences were aligned using MUSCLE, as implemented in MEGA-11, ModelFinder was used for model selection, and IQ-TREE was employed to construct the phylogenetic tree using the maximum likelihood (ML) method, with bootstrap support values tested using 1,000 replicates.

## 3 Results

### 3.1 Analysis of potential zoonotic bacterial pathogens

Using the “Global Pathogen Database (gcPathogen)” developed by the National Microbiology Data Center (NMDC) of the Institute of Microbiology, Chinese Academy of Sciences,^2^ we identified 49 species across 24 genera with zoonotic potential out of 158 species from 84 bacterial genera (Table 1). These zoonotic bacterial genera exhibited substantial variability in prevalence and total read counts. High detection rates and abundances were observed in *Acinetobacte*r, *Escherichia*, *Klebsiella*, *Ralstonia*, *Rothia*, and *Streptococcus*. The prevalence and relative abundance were calculated for species with a prevalence greater than 10/30. The results were as follows: *E. coli* had a prevalence of 30/30 and a relative abundance of 1.59–98.09%; *Acinetobacter baumannii* had a prevalence of 28/30 and a relative abundance of 0.59–60.45%; *Afipia broomeae* had a prevalence of 24/30 and a relative abundance of 0.005–3.72%; *K. pneumoniae* had a prevalence of 22/30 and a relative abundance of 0.57–62.06%; *Ralstonia pickettii* had a prevalence of 17/30 and a relative abundance of 0.033–3.32%; and *Rothia dentocariosa* had a prevalence of 11/30 and a relative abundance of 0.26–12.3%.

**Table 1:** Composition of microorganisms with zoonotic potential in raccoon dog lungs.

Genus	Species	Prevalence rate	Relative abundance range
Actinomyces	*Actinomyces massiliensis*	2/30	0.24–0.41
	*Actinomyces naeslundii*	1/30	0.32
	*Actinomyces oris*	1/30	0.36
	*Actinomyces oris E*	1/30	0.25
Brevibacillus	*Brevibacillus parabrevis*	3/30	0.37–2.82
Enterococcus	*Enterococcus faecalis*	3/30	0.73–6.47
Veillonella_	*Veillonella atypica*	1/30	4.13
Cutibacterium	*Cutibacterium acnes*	6/30	0.03–1.55
Bacteroides	*Bacteroides pyogenes*	1/30	0.086
Prevotella	*Prevotella histicola*	7/30	0.29–5.72
	*Prevotella jejuni*	1/30	1.54
	*Prevotella multiformis*	1/30	0.08
	*Prevotella nigrescens*	2/30	0.29–0.73
	*Prevotella oris*	1/30	0.15
Citrobacter	*Citrobacter braakii*	1/30	0.64
Escherichia	*Escherichia coli*	30/30	1.59–98.09
	*Escherichia albertii*	1/30	0.03
	*Escherichia fergusonii*	1/30	0.01
	*Escherichia marmotae*	1/30	0.02
Aeromonas	*Aeromonas caviae*	1/30	1.6
	*Aeromonas dhakensis*	1/30	0.62
	*Aeromonas media*	1/30	0.18
Acinetobacter	*Acinetobacter baumannii*	28/30	0.59–60.45
	*Acinetobacter guillouiae*	1/30	0.17
	*Acinetobacter soli*	1/30	0.89
Klebsiella	*Klebsiella aerogenes*	1/30	2.07
	*Klebsiella michiganensis*	1/30	1.07
	*Klebsiella pneumoniae*	22/30	0.57–62.06
Ralstonia	*Ralstonia pickettii*	17/30	0.033–3.32
	*Ralstonia pickettii B*	9/30	0.004–1.23
Rothia	*Rothia dentocariosa*	11/30	0.26–12.3
	*Rothia mucilaginosa A*	1/30	1.14
Chlamydophila	*Chlamydophila abortus*	3/30	10.26–27.1
Streptococcus	*Streptococcus agalactiae*	1/30	6.12
	*Streptococcus alactolyticus*	2/30	2.08–3.47
	*Streptococcus lactarius*	6/30	0.22–5.54
	*Streptococcus lutetiensis*	1/30	8.66
	*Streptococcus minor*	2/30	0.23–1.38
Lactococcus	*Lactococcus garvieae*	2/30	1.87–2.48
	*Lactococcus lactis*	1/30	5.10
Afipia	*Afipia broomeae*	24/30	0.005–3.72
Salmonella	*Salmonella enterica*	4/30	0.02–13.47
Proteus	*Proteus mirabilis*	1/30	6.07
Enterobacter	*Enterobacter hormaechei A*	2/30	0.02–3.7
	*Enterobacter kobei*	1/30	1.1
	*Enterobacter quasihormaechei*	1/30	0.54
Bordetella	*Bordetella pertussis*	1/30	2.5
Pseudomonas B	*Pseudomonas B oryzihabitans*	1/30	0.45
Bacteroides	*Bacteroides pyogenes*	1/30	0.09

The information in this column is based on the “Global Pathogen Database (gcPathogen)” developed by the National Microbiology Data Center (NMDC) of the Institute of Microbiology, Chinese Academy of Sciences (https://nmdc.cn/gcpathogen/).

### 3.2 Fungal composition of the lung tissue

At the phylum level, Ascomycota were detected in all samples, with relative abundances ranging from 0.07 to 23.17%. Basidiomycota were observed in only one sample (0.02%). At the genus level, *Talaromyces*, *Pichia*, and *Fusarium* were identified in all samples, with relative abundances of 0.01–2.82%, 0.06–19.95%, and 0.005–1.65%, respectively. *Moesziomyces* was detected in only one sample (0.02%). At the species level, the abundance patterns of *T. rugulosus*, *P. inconspicua*, *F. oxysporum*, and *Moesziomyces antarcticus* aligned with their respective genus-level distributions.

### 3.3 Bacterial and fungal diversity in lung tissue samples

**Figure 1:**
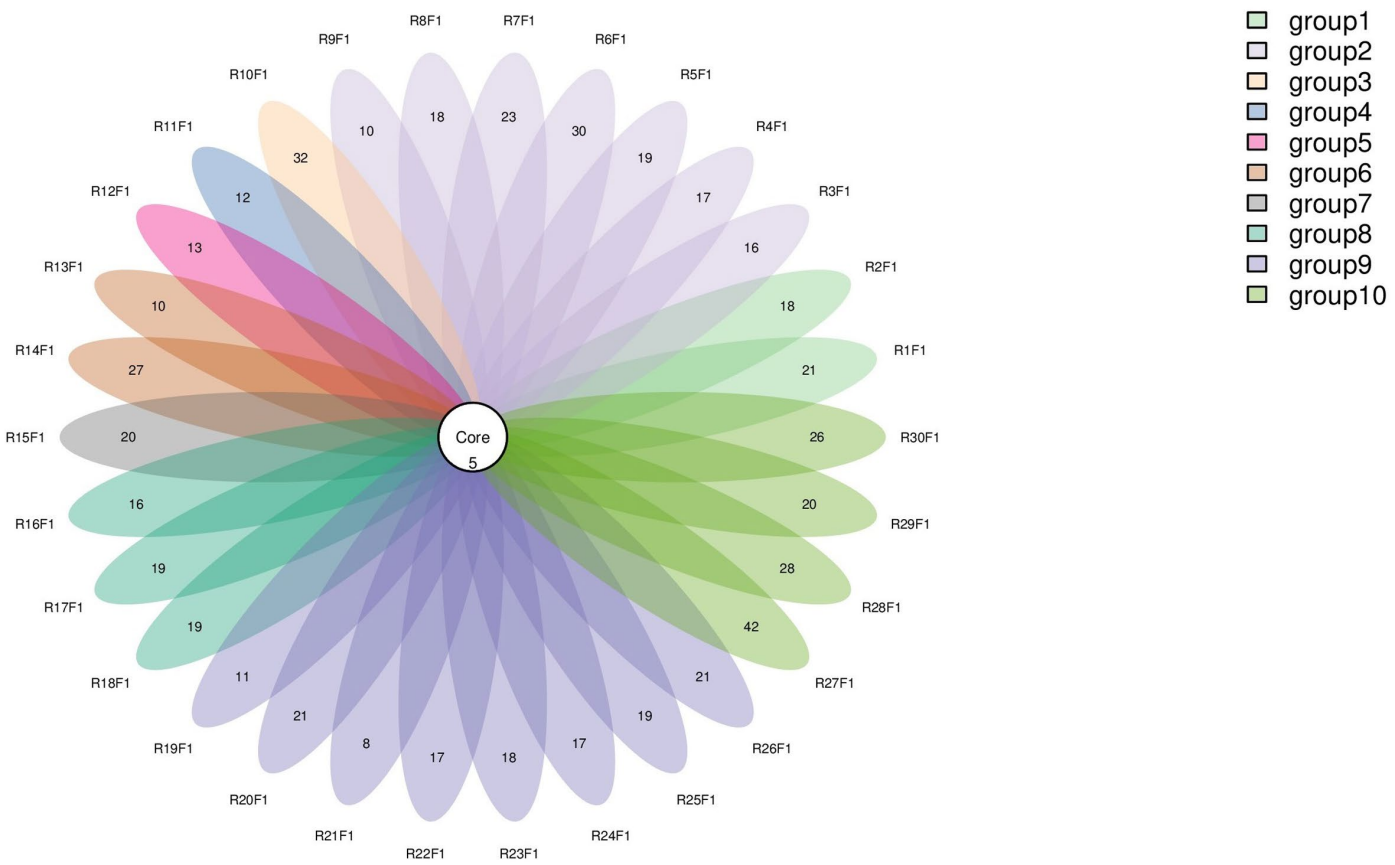
Venn diagram of species distribution. Each oval (labeled with an RF identifier) corresponds to one animal.

**Figure 2:**
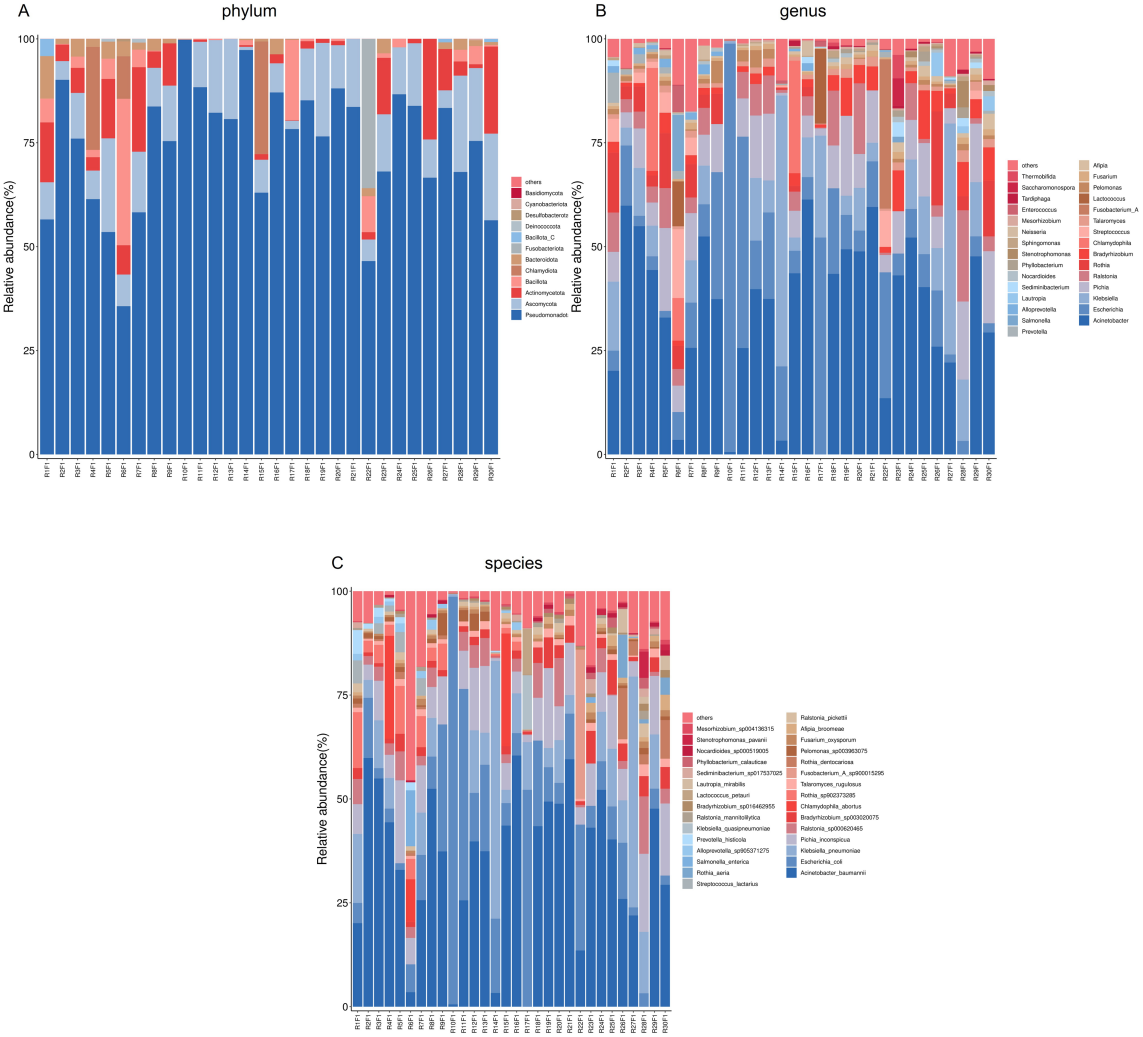
Microbial community composition of lung tissue samples. **(A)** Bar plot of phylum-level community structure across samples; **(B)** Bar plot of the top 30 genera; **(C)** Bar plot of the top 30 species.

**Figure 3:**
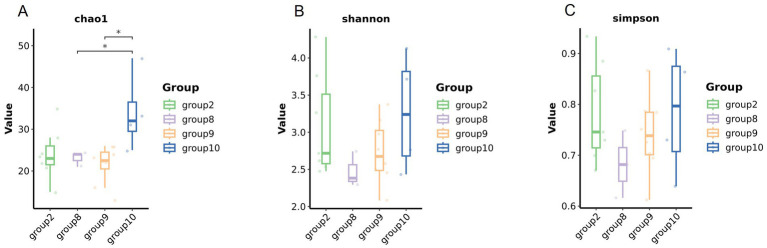
Microbial diversity of the lung tissue samples in different groups. Comparison of the alpha diversity among groups (**A** Chao1 index, **B** Shannon index, and **C** Simpson index).

Numbers of 2bRAD reads before and after filtering, including raw, enzyme-digested, and clean reads, are presented in Supplementary Table S2. A total of 158 microbial species were identified across 30 samples. A Venn diagram revealed the number of species shared by different sample groups, showing that five species were common to all 30 samples: *Escherichia coli* (1.59–98.09%), *Pichia inconspicua* (0.06–19.95%), *Bradyrhizobium sp003020075* (0.04–8.31%), *Talaromyces rugulosus* (0.01–2.82%), and *Fusarium oxysporum* (0.005–1.65%) (Figure 1). Microbial *α*-diversity and community composition analyses are shown in Figure 2. At the phylum level, dominant taxa were Pseudomonadota (35.67–99.77%), Ascomycota (0.07–23.17%), and Actinobacteria (0–24.21%). At the genus level, the dominant genera were *Acinetobacter* (0–61.33%), *Escherichia* (1.59–98.26%), and *Klebsiella* (0–65.2%). The five most abundant species were *Acinetobacter baumannii* (0–60.45%), *E. coli* (1.59–98.09%), *Klebsiella pneumoniae* (0–62.06%), *P. inconspicua* (0.06–19.95%), and *Ralstonia sp000620465* (0–13.82%). Alpha diversity was assessed in groups with over three lung tissue samples (Groups 2, 8, 9, and 10). No significant differences were observed in the Shannon or Simpson indices among the groups, except for a significant difference in the Chao1 index between Groups 9 and 10 as well as between Groups 8 and 10 (Figure 3; Table 2).

**Table 2:** Significance test of chao1, Shannon and Simpson diversity indices between different groups.

Groups		*p*-value		
		Chao1	Shannon	Simpson
2	8	1	0.18	0.27
	9	0.68	0.4	0.54
	10	0.073	0.93	1
8	9	0.92	0.28	0.28
	10	0.05	0.11	0.4
9	10	0.021^*^	0.46	0.57

“*”indicates a statistically significant difference (*p* < 0.05).

### 3.4 Beta diversity of bacterial and fungal communities across farms

**Figure 4:**
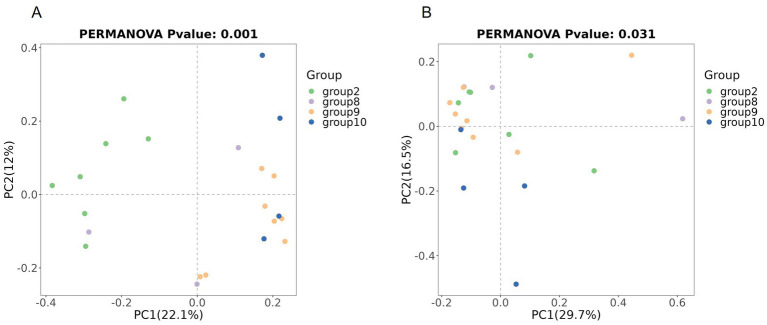
Comparison of beta diversity between the four groups based on PCoA. Comparison of the beta diversity among groups based on the Binary Jaccard distance **(A)**, and Bray–Curtis distance **(B)**.

Beta diversity compares microbial differences across multiple groups. Permanova analysis revealed the overall microbial composition was significantly different (*p* = 0.001, *p* = 0.031) among groups based on the Binary Jaccard distance and Bray–Curtis distance (Figures 4A,B).

A statistical comparison of median differences using the Kruskal-Wallis H test was performed for Groups 2, 8, 9, and 10 at the phylum, genus, and species levels (Supplementary Table S3).

Phylum level: No significant differences were detected in Pseudomonadota, Ascomycota, Actinobacteria, Bacillota, or Chlamydiota. However, Bacteroidota showed a highly significant difference between Groups 2 and 9 (*p* = 0.003), with higher abundance in Group 2.

Genus level: No significant differences were detected among *Acinetobacter*, *Klebsiella*, or *Escherichia*. *Rothia* was significantly more abundant in Group 2 than in Group 9 (*p* = 0.034).

Species level: No significant differences were observed for *Acinetobacter baumannii*, *E. coli*, or *K. pneumoniae*. However, *Rothia sp902373285* was significantly higher in Group 2 vs. Groups 9 (*p* = 0) and 10 (*p* = 0.006). *Streptococcus lactarius* was significantly more common in Group 2 than in Group 9 (*p* = 0.033). *Alloprevotella sp905371275* was significantly elevated in Group 2 vs. Group 9 (*p* = 0.005) and Group 10 (*p* = 0.035). *Prevotella histicola* was significantly more common in Group 2 than in Group 9 (*p* = 0.033). *Lautropia mirabilis* was significantly more common in Group 2 than in Group 10 (*p* = 0.024). Compared with that in Group 9, the abundance of *Phyllobacterium calauticae* in Group 2 was significantly different (*p* = 0.008).

### 3.5 Viral metagenomics analysis of lung tissue

#### 3.5.1 Raw data quality control results

Numbers of sequencing reads during virome sequencing quality control are shown in Supplementary Table S4. For Library 1, 58,512,218 raw reads were obtained, reduced to 56,042,140 reads after filtering, and further decreased to 28,021,070 reads after contaminant removal. Library 2 initially generated 75,895,168 raw reads, which were filtered to 69,573,170 reads and reduced to 34,786,585 reads after contaminant removal. Library 3 started with 65,659,632 raw reads, which were filtered to 61,543,548 reads; 30,771,774 reads were retained after contaminant removal.

#### 3.5.2 Taxonomic annotation results at the family level

**Figure 5:**
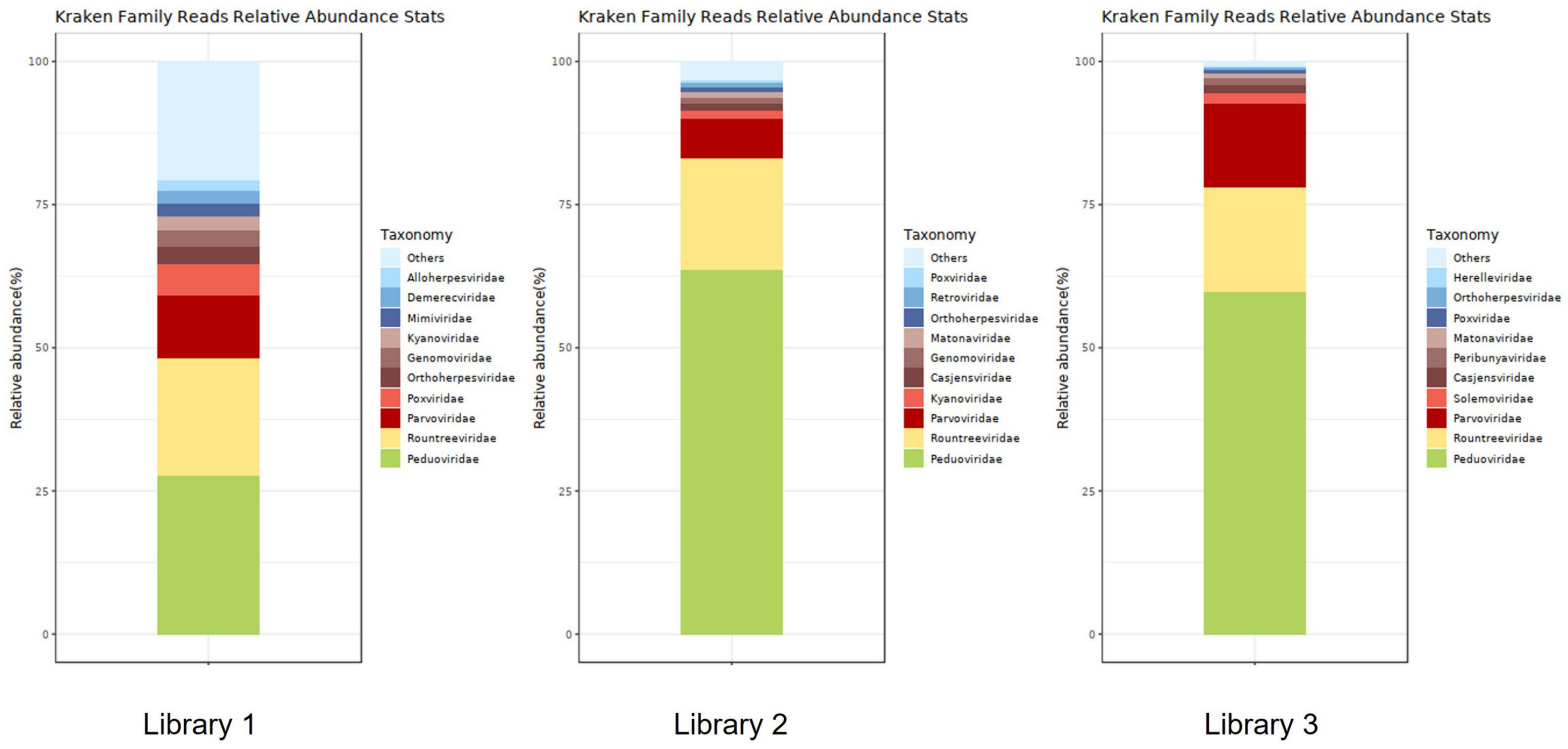
Stacked plot of annotated relative abundance at the family level of viruses in lung tissue.

Figure 5 shows the annotation results of the sequences at the viral family level. According to Table 3, the average relative abundances of the top ten viral families across the three libraries were calculated as follows: *Peduoviridae* (50.4%), *Rountreeviridae* (19.44%), *Parvoviridae* (10.82%), *Poxviridae* (2.1%), *Genomoviridae* (1.92%), *Orthoherpesviridae* (1.45%), *Kyanoviridae* (1.37%), *Demerecviridae* (1.12%), *Retroviridae* (1.11%), and *Casjensviridae* (1.02%).

**Table 3:** Distribution of viral sequences across different viral families in lung tissue.

Name	Library 1		Library 2		Library 3	
	Reads	Fraction	Reads	Fraction	Reads	Fraction
*Peduoviridae*	2,862	0.27786	16,049	0.63586	5,746	0.59842
*Rountreeviridae*	2,116	0.20544	4,935	0.19552	1751	0.18236
*Parvoviridae*	1,131	0.10981	1749	0.06929	1,397	0.14549
*Kyanoviridae*	262	0.02544	363	0.01438	12	0.00125
*Casjensviridae*	65	0.00631	298	0.01181	119	0.01239
*Genomoviridae*	281	0.02728	283	0.01121	0	0
*Matonaviridae*	25	0.00243	245	0.00971	90	0.00937
*Orthoherpesviridae*	322	0.03126	204	0.00808	41	0.00427
*Retroviridae*	154	0.01495	183	0.00725	0	0
*Demerecviridae*	225	0.02184	16	0.00063	0	0
*Alloherpesviridae*	196	0.01903	62	0.00246	0	0
*Mimiviridae*	228	0.02214	65	0.00258	20	0.00208
*Poxviridae*	550	0.05340	101	0.00400	55	0.00573
*Solemoviridae*	43	0.00417	27	0.00107	192	0.02000
*Peribunyaviridae*	56	0.00544	28	0.00111	118	0.01229

#### 3.5.3 Taxonomic annotation results at the species level

**Figure 6:**
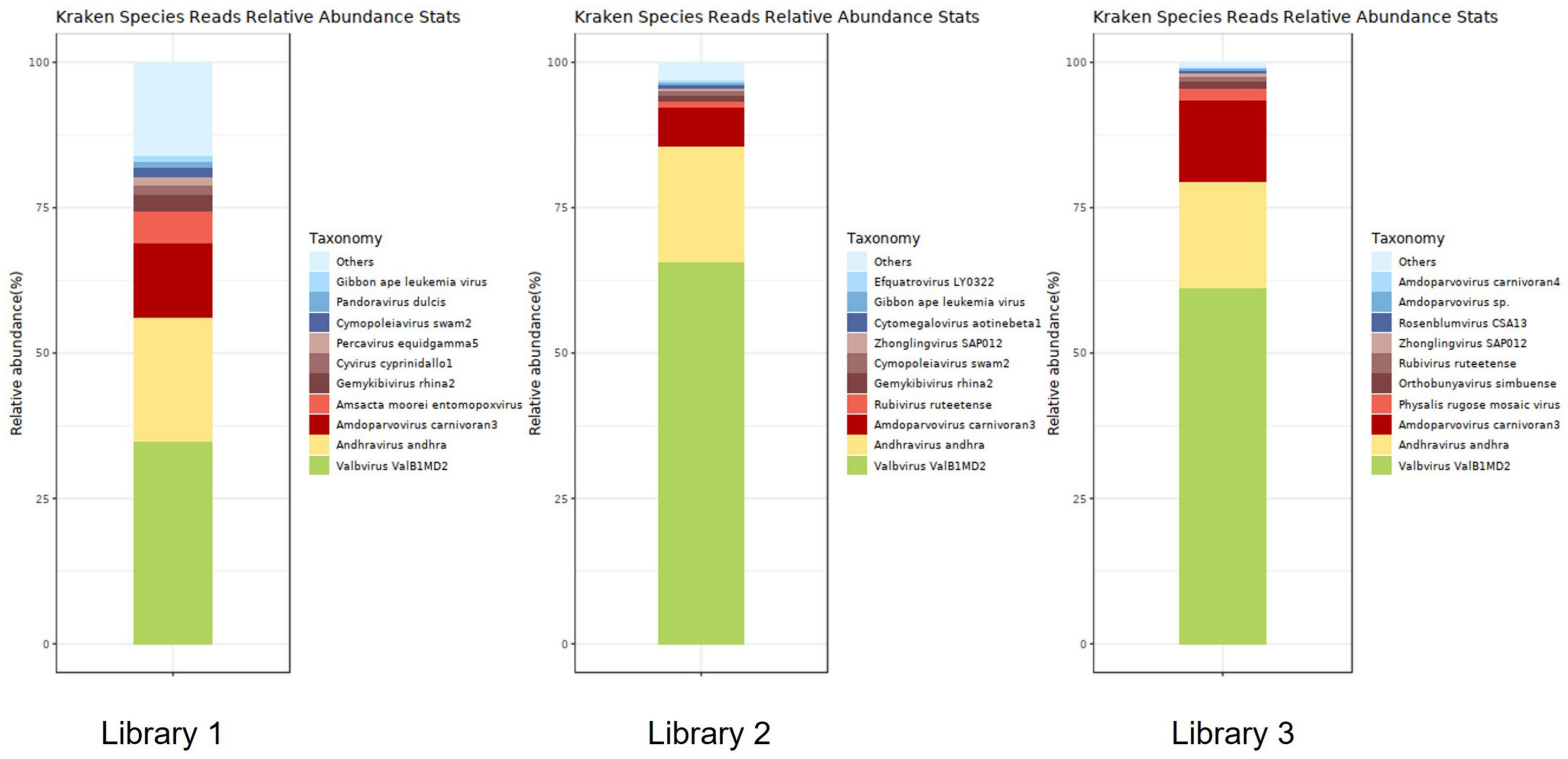
Stacked plot of annotated relative abundance of viral species levels in raccoon dog lung tissue.

Figure 6 shows the annotation results for the sequences at the viral species level. According to Table 4, the average relative abundances of the top ten viral species in the lung tissue at the species level were calculated as follows: *Valbvirus ValB1MD2* (53.92%), *Andhravirus andhra* (19.81%), *Amdoparvovirus carnivoran3* (11.19%), *Amsacta moorei entomopoxvirus* (1.81%), *Gemykibivirus rhina2* (1.26%), *Rubivirus ruteetense* (0.76%), *Cymopoleiavirus swam2* (0.75%), *Physalis rugose mosaic virus* (0.73%), *Percavirus equidgamma5* (0.63%), and *Cyvirus cyprinidallo1* (0.59%).

**Table 4:** Distribution of viral sequences across different viral species in lung tissue.

Name	Library 1		Library 2		Library 3	
	Reads	Fraction	Reads	Fraction	Reads	Fraction
*Valbvirus ValB1MD2*	2,843	0.34926	16,031	0.65628	5,746	0.61212
*Andhravirus andhra*	1728	0.21229	4,882	0.19986	1711	0.18227
*Percavirus equidgamma5*	121	0.01486	25	0.00102	29	0.00309
*Cyvirus cyprinidallo1*	140	0.01720	11	0.00045	0	0
*Amdoparvovirus carnivoran3*	1,041	0.12789	1,652	0.06763	1,316	0.14019
*Gemykibivirus rhina2*	231	0.02838	230	0.00942	0	0
*Amsacta moorei entomopoxvirus*	441	0.05418	0	0	0	0
*Cymopoleiavirus swam2*	120	0.01474	192	0.00786	0	0
*Pandoravirus dulcis*	91	0.01118	39	0.00160	0	0
*Rubivirus ruteetense*	25	0.00307	245	0.01003	90	0.00959
*Zhonglingvirus SAP012*	18	0.00221	131	0.00536	49	0.00522
*Physalis rugose* mosaic virus	10	0.00123	16	0.00066	189	0.02013
*Orthobunyavirus simbuense*	28	0.00344	13	0.00053	116	0.01236

#### 3.5.4 A virus belonging to the genus *Amdoparvovirus*

**Figure 7:**
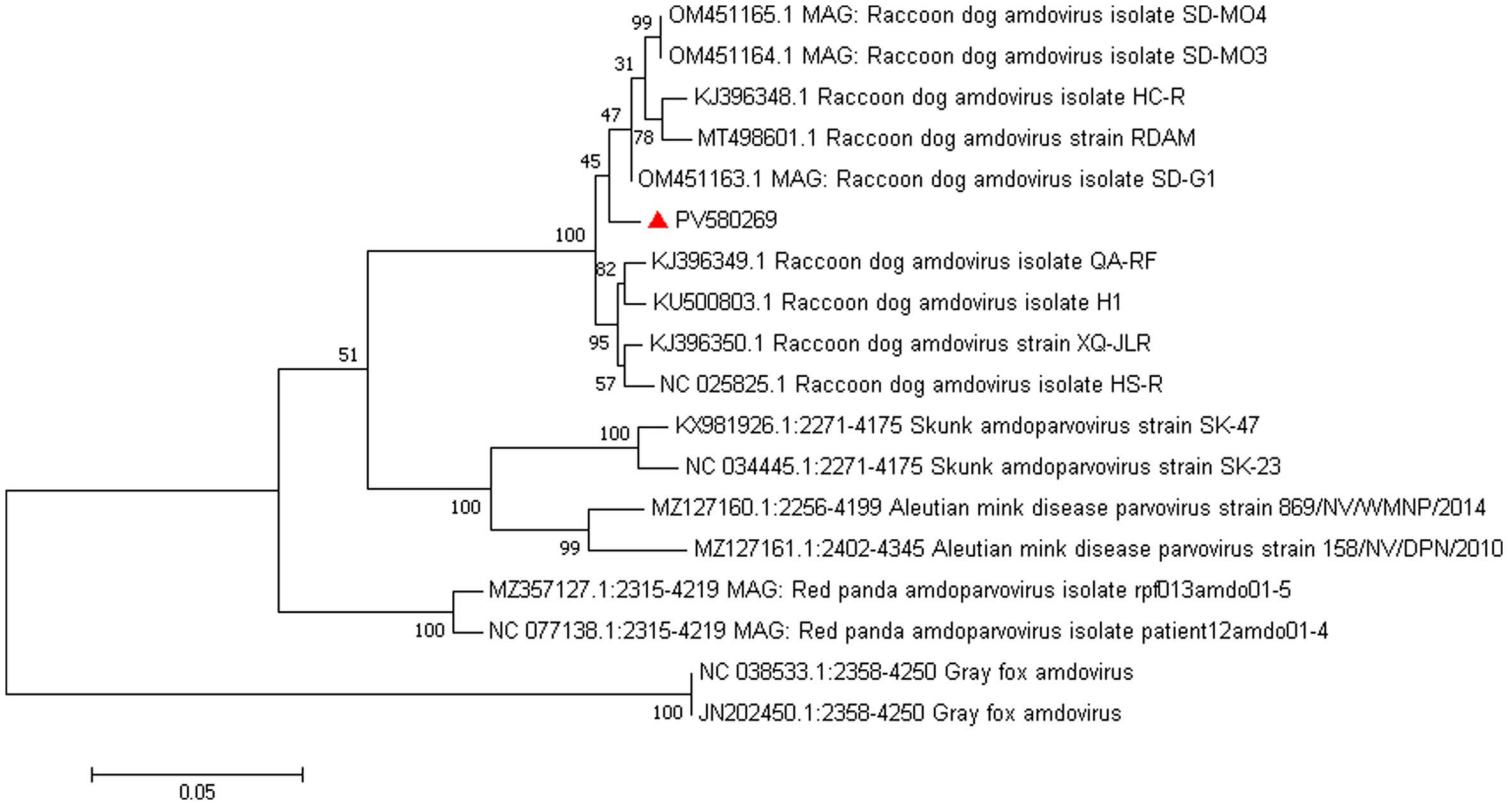
Phylogenetic tree analysis of the VP2 gene in raccoon dog *Amdoparvovirus*. The VP2 identified in this study was marked with a red triangle.

**Figure 8:**
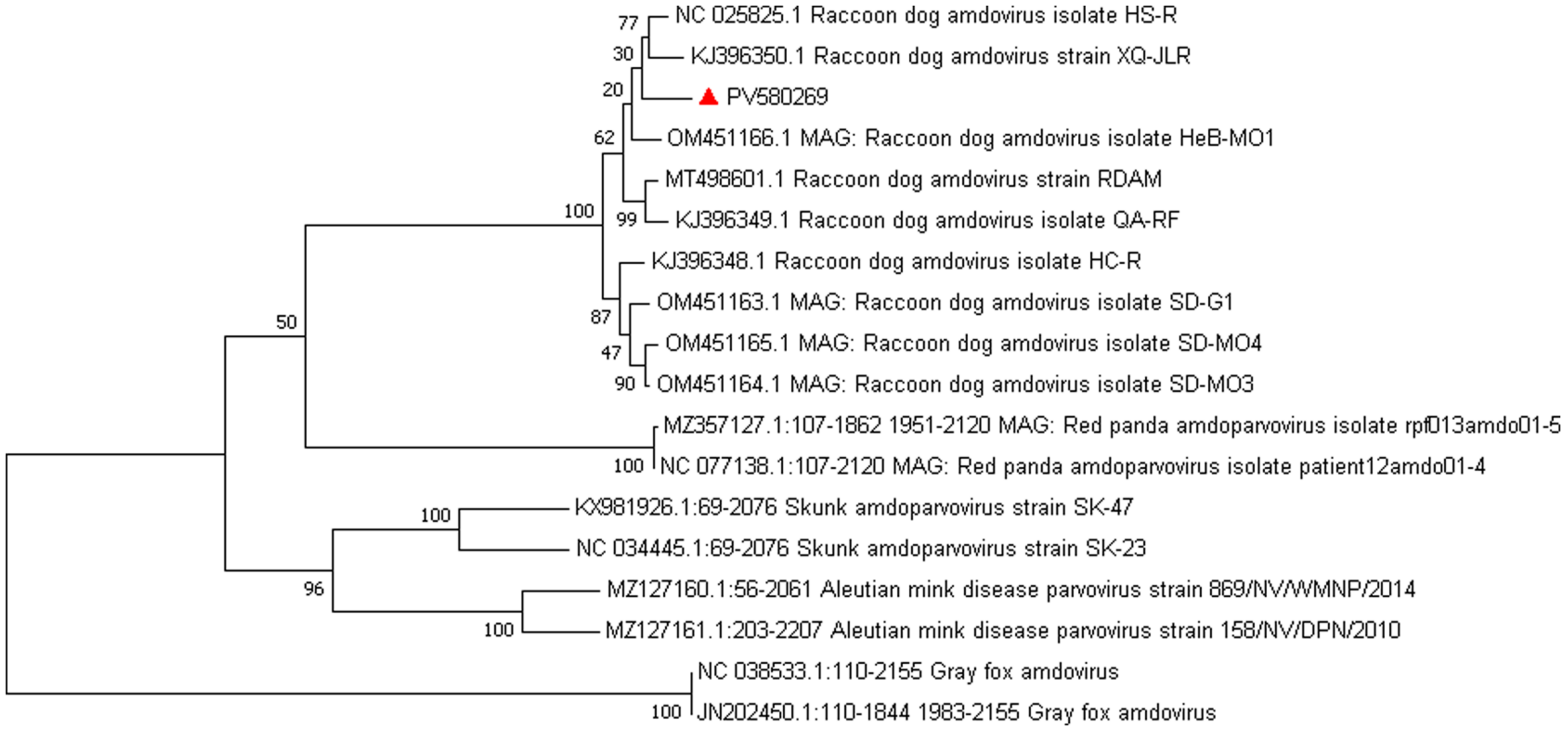
Phylogenetic tree analysis of the NS1 gene in raccoon dog *Amdoparvovirus*. The NS1 identified in this study was marked with a red triangle.

*Amdoparvovirus* sequences were detected in all three libraries. Using the Align/Assemble function of Geneious 11.1.2 in Library 3, a nearly complete *Amdoparvovirus* genome was obtained, with a length of 4,635 bp, containing three nonstructural proteins and two structural proteins. The nucleotide sequence of the genome was submitted to GenBank (accession number: PV580269). Phylogenetic analysis of NS1 and VP2 sequences, including 17 references from the five recognized *Amdoparvovirus* species within the genus *Amdoparvovirus*: *Raccoon dog amdoparvovirus*, *Skunk amdoparvovirus*; *Aleutian mink disease parvovirus* (AMDV), *Red panda amdoparvovirus*, and *Gray fox amdoparvovirus 3*, resolved five major clades. Four clades aligned with the known species, while the fifth consisted of our sequences. Notably, our samples clustered into a distinct subclade within a larger clade that includes sequences from raccoon dogs (Figures 7, 8). The assembled contigs were compared to the virus-NT database using BLAST (V2.10.0+). NCBI BLASTn analysis revealed that the NS1 nucleotide sequences of our samples shared the highest identity (97.82%) with those of the strain HS-R (GenBank no. NC_025825.1), covering a length of 1856/1923 nt, whereas the VP2 sequence showed the highest identity (98.71%) with strain SD-G1 (GenBank no. OM451163.1), covering a length of 1716/1756 nt. Strain HS-R was identified in 2013 from raccoon dog spleen tissue samples collected during a disease outbreak on a farm in Jilin Province, China (Shao XQ, et al., 2014), while strain SD-G1 was identified in 2021 from raccoon dog tissue samples collected in Hebei and Shandong Provinces, China (He et al., 2022). Compared to these strains, our sample presented 44 and 25 nucleotide sequence variations in the NS1 and VP2 regions, respectively.

#### 3.5.5 Identification of a novel *Anelloviridae* virus in raccoon dogs

**Figure 9:**
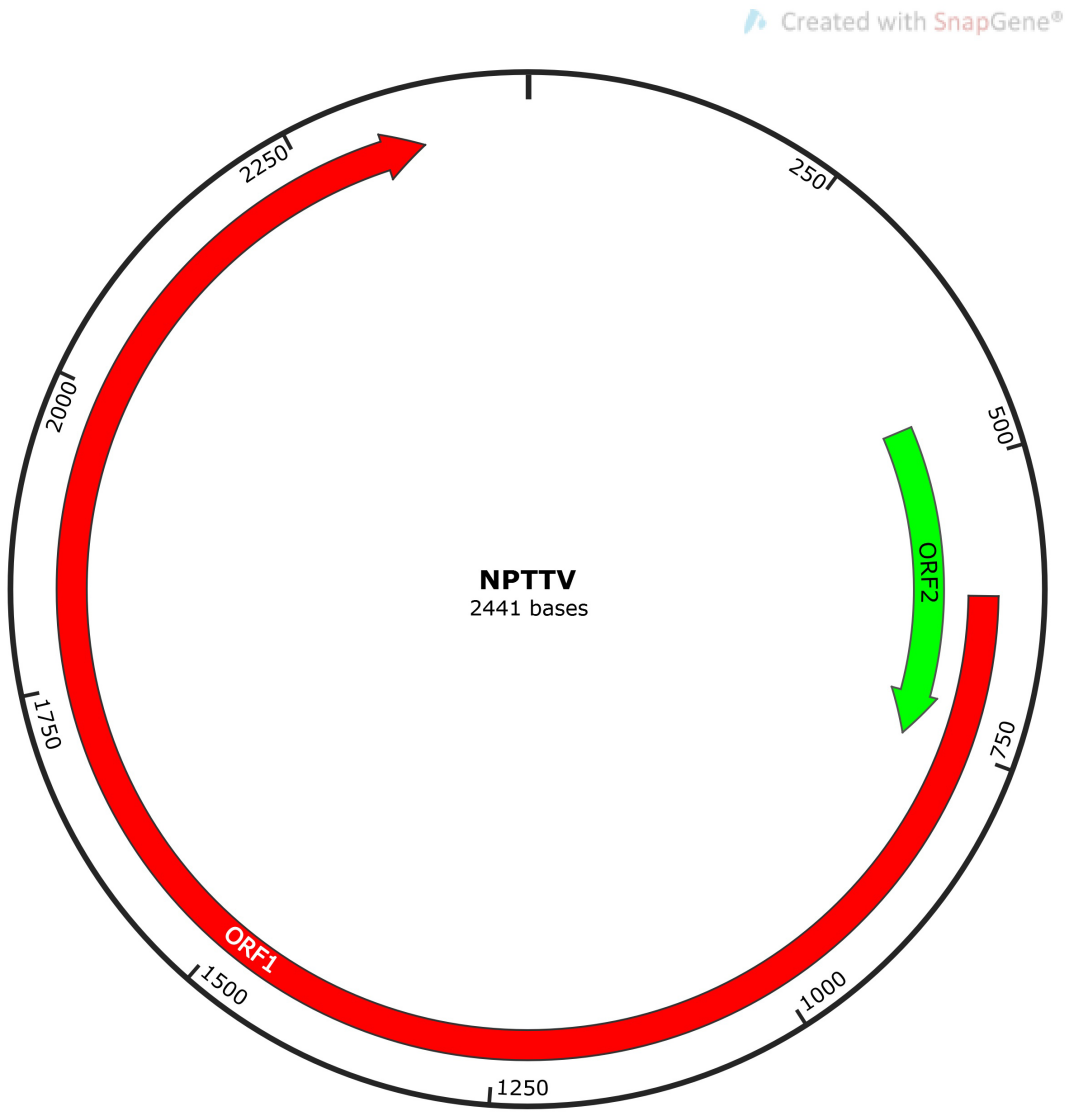
Genomic structure schematic diagram of NPTTV.

**Figure 10:**
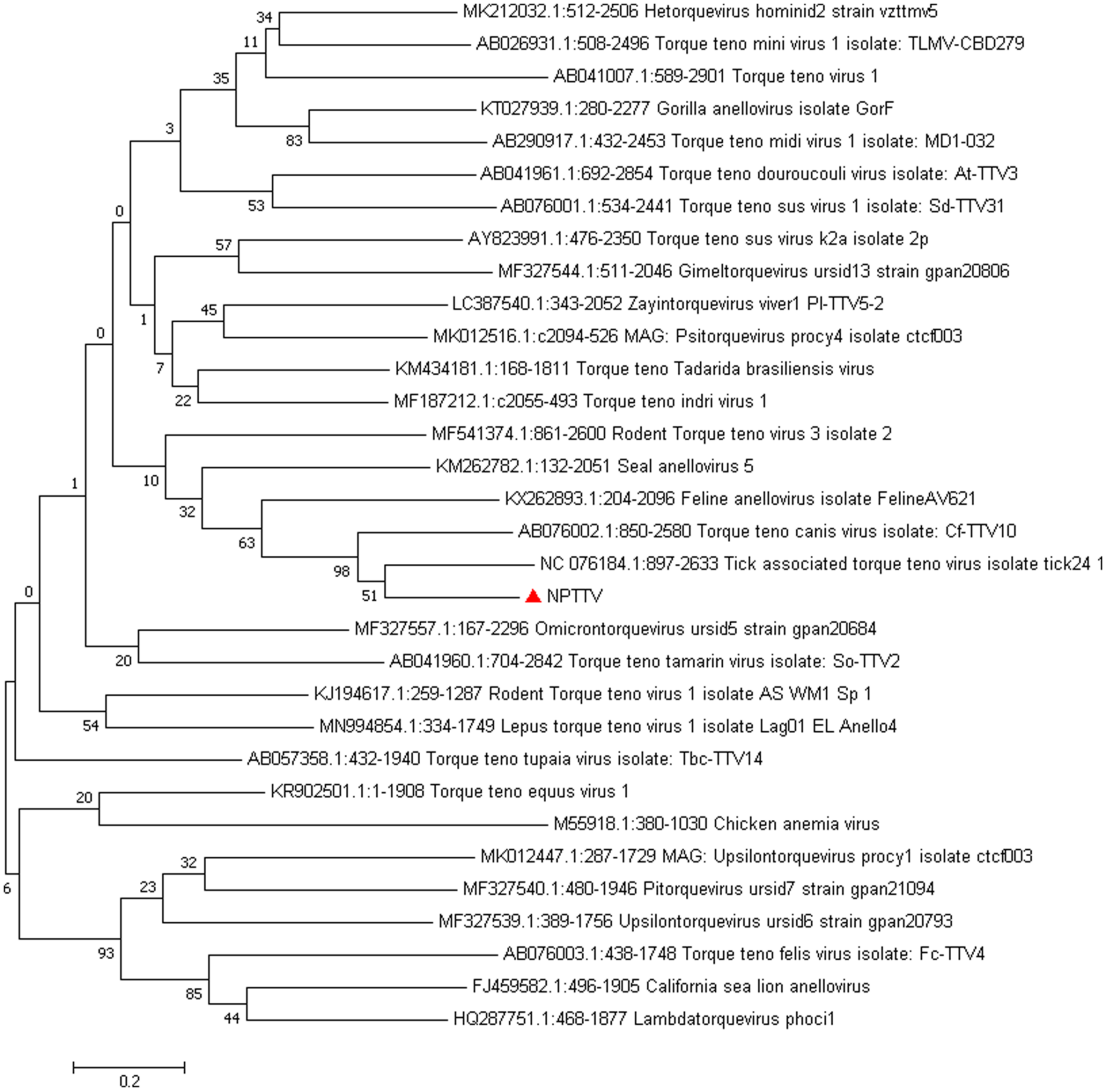
Phylogenetic tree analysis of the ORF1 gene in raccoon dog TTV. The ORF1 identified in this study was marked with a red triangle.

*Torque Teno Virus* (TTV) sequences were detected in two libraries. A nearly complete genome, designated NPTTV, was successfully assembled with a length of 2,441 bp (GenBank accession no. PV693371). The gene structure is illustrated in Figure 9 and contains two open reading frames (ORFs). Phylogenetic analysis of ORF1 revealed that the nucleotide sequences of NPTTV were grouped within a clade of tick-associated TTV isolate tick24_1 (GenBank accession: NC_076184.1) (Figure 10), BLAST results of the ORF1 sequence showed only 39 nucleotides with a similarity of 92%. Additionally, BLAST results of the 470 bp untranslated region (UTR) sequence revealed 83.76% sequence identity with the tick-associated TTV isolate tick24_1.

#### 3.5.6 Other viruses detected as short sequence fragments

In Library 1, we detected partial sequences with similarity to coronaviruses of SARS-CoV-2 (210 bp) with an alignment length of 103 bp. BLAST results revealed 81.55% identity with a segment of the genome of the *severe acute respiratory syndrome coronavirus 2* isolate Wuhan-Hu-1 (GenBank accession: OU471411.1). In Library 2, we identified partial sequences of raccoon dog TTV (400 bp) with an alignment length of 214 bp. BLAST analysis revealed 89.25% identity with a region of the complete genome of raccoon dog TTV 2 Raccoon dog/Japan/Rac_Fe2/2022 DNA (GenBank accession: LC743590.1). Additionally, partial sequences of *human betaherpesvirus 6* (374 bp in length) were detected in Library 2, with an alignment length of 380 bp and 81.55% identity to a segment of the *human betaherpesvirus 6* strain HP8H1 partial genome (GenBank accession: KY315527.2).

## 4 Discussion

Raccoon dogs are known carriers of various pathogens, yet their pulmonary microbiota remains underreported. In contrast, healthy human lungs have been reported to host Firmicutes, Proteobacteria, and Bacteroidetes (phylum level) and *Pseudomonas*, *Streptococcus*, *Prevotella*, *Fusobacterium*, and *Veillonella* (genus level) (Maddi et al., 2019). Fungal communities in human lungs are primarily composed of Ascomycota and Basidiomycota at the phylum level, with *Candida* and *Pichia* dominating at the genus level (Wu et al., 2022). The prevalence of Pseudomonadota and Ascomycota in raccoon dogs may reflect shared environmental exposure between humans and raccoon dogs, as backyard breeding practices often lack spatial separation between living and farming areas, increasing inhalation of airborne microorganisms. Notably, Pseudomonadota and Ascomycota are among the most common microbial taxa in the air (Sharma Ghimire et al., 2022). The composition of respiratory microbiota exhibits substantial variation among several animal species, including pigs (Huang et al., 2019; Li et al., 2021; Jiang et al., 2019), sheep (Miao et al., 2023), and dogs (Vientós-Plotts et al., 2019; Ericsson et al., 2016). Overall, the microbial profiles of raccoon dogs are similar to those of canines, with Proteobacteria and Actinobacteria as dominant phyla and *Acinetobacter* as the predominant genus, likely attributable to their taxonomic classification within the family Canidae (Fastrès et al., 2019).

In this sequencing study, we identified numerous potentially pathogenic and opportunistic bacteria in raccoon dog lungs, including 49 species across 24 genera with potential zoonotic risks. Several exhibited high relative abundance (0.01–98.09%) and prevalence (≥10/30), including *Escherichia coli* (Wang et al., 2025) (pathogenic strains can cause diarrhea, pneumonia, and more), *A. baumannii* (Al-Hassan and Al-Madboly, 2020) (a major nosocomial and opportunistic pathogen), and *Klebsiella pneumoniae* (associated with pneumonia and sepsis), *Ralstonia* species (Jin et al., 2025; Sebastian et al., 2025), *Afipia broomeae* (Brenner et al., 1991), *Rothia dentocariosa* (Zheng et al., 2024). Notably, *Bordetella pertussis*, the gram-negative causative agent of whooping cough, was detected in one tissue sample (2.5% abundance). *Salmonella enterica*, a well-known zoonotic pathogen (Chiu et al., 2004), was detected in four samples (0.02–13.47%). *Chlamydophila abortus,* identified in three samples (10.26–27.1%), is known to cause reproductive losses in livestock and flu-like symptoms such as fever, chills, headache, and myalgia in humans, with severe cases potentially progressing to respiratory diseases such as pneumonia and bronchitis (Albini et al., 2023). Six *Streptococcus* species associated with humans were detected, including *Streptococcus agalactiae* (Group B *Streptococcus*), identified in one sample (6.12%), a pathogen associated with bovine mastitis and streptococcosis in cattle and fish, respectively (Crestani et al., 2024). In humans, it poses significant risks to newborns and immunocompromised individuals, potentially causing fatal infections and long-term sequelae (Yuan et al., 2021). *Staphylococcus pseudintermedius*, detected in one sample (1.4%), is transmitted primarily to humans through close contact with dogs, and its pathogenic mechanisms resemble those of *Staphylococcus aureus* (Lynch and Helbig, 2021). These findings suggest that raccoon dogs may serve as reservoirs for multiple zoonotic pathogens, posing potential public health risks.

Using viral metagenomics, we annotated 93,579,429 reads from three sequencing libraries as viral sequences. Dominant families included *Peduoviridae*, *Rountreeviridae*, and *Parvoviridae*. The most abundant viral species were *Valbvirus ValB1MD2*, *Andhravirus andhra*, and *A. carnivoran3*. These findings indicate that bacteriophages dominate the raccoon dog lung virome and may interact with prevalent bacterial hosts such as *Acinetobacter baumannii*, *E. coli*, and *K. pneumoniae*. In addition to bacteriophages, *A. carnivoran3* exhibited relatively high abundance (>80%). We also identified novel strains of raccoon dog *Amdoparvovirus* and TTV, and detected partial short genomic fragments of SARS-CoV-2, raccoon dog TTV, and human betaherpesvirus 6. However, the exceedingly short read lengths and low abundance prevent definitive confirmation of these viruses or assessment of their biological significance in raccoon dogs. These signals could potentially originate from laboratory contamination or environmental exposure rather than genuine infection. Further targeted assays would be necessary to validate their presence and significance.

Raccoon dog and arctic fox amdoparvovirus (RFAV) is an amdoparvovirus that naturally infects raccoon dogs and blue foxes, genetically distinct from Aleutian mink disease virus (AMDV) (Shao et al., 2014). Its genome is approximately 4,800 nucleotides in length and contains two large ORFs that encode structural proteins VP1 and VP2, as well as non-structural proteins NS1, NS2, and NS3. In this study, NS1 sequences showed 97.82% identity with strain HS-R (identified in 2013 in Jilin Province), while VP2 showed 98.71% identity with strain SD-G1 (identified in 2021 in Hebei and Shandong). The high homology with strains HS-R and SD-G1, which were identified in different years and provinces, confirms the circulation of this viral species in the region. The genetic stability observed across time and geography warrants further investigation.

TTV was first detected in 1997 in a patient with unexplained posttransfusion hepatitis (Nishizawa et al., 1997) and has since been identified in humans and other mammals, including nonhuman primates, livestock, and wildlife (Manzin et al., 2015). TTV is a single-stranded, negative-sense, circular DNA virus with a genome length of approximately 2.1–3.9 kb. The genome contains three ORFs encoding proteins VP1, VP2, and VP3, with structural variation across genera. Taxonomic classification of anelloviruses is based on nucleotide sequence similarity of ORF1 with cut-off values of 44 and 65%, respectively, for genus and species (Biagini et al., 2011).

In this study, BLAST analysis showed that the ORF1 sequence of NPTTV had only 39 nucleotides with 92% similarity to the tick-associated TTV isolate tick24_1, the 470 bp partial UTR sequence revealed 83.76% sequence identity with it. While the entire ORF1 sequence of NPTTV shows 42.9 and 50.3% similarity to the tick-associated TTV isolate tick24_1 and the Torque teno canis virus isolate *Cf*-TTV10, respectively. Based on the guidelines for classification for the family *Anelloviridae*, NPTTV from this study belongs to the genus *Thetatorquevirus* is a putative new species. It is likely a recombinant chimera, with its ORF1 region originating from an unknown source and its UTR region derived from a tick TTV-like ancestor.

This study offers valuable preliminary insights into pulmonary pathogens in raccoon dogs but exhibits several limitations. The absence of healthy control groups limits the interpretation of our microbiological findings. In the absence of such a comparison, it is difficult to determine whether the microorganisms that have been detected are true pathogens, part of the normal commensal flora, or environmental contaminants. Methodologically, viral metagenomics’ low sensitivity may overlook low-abundance viruses. Notably, the absence of histopathological correlation and pathogen isolation means that we cannot definitively establish a causal relationship between the identified microbes and the observed pneumonia. Future studies employing histopathology, microbial culture, and *in situ* hybridization are essential to confirm the tissue tropism and pathogenic role of these microbes.

In summary, this study employed viral metagenomics and 2bRAD-M to investigate viral, bacterial, and fungal carriage in raccoon dog lung tissues. The results revealed substantial amounts of potentially pathogenic and opportunistic pathogens, underscoring raccoon dogs as potential carriers posing public health risks. Notably, a novel raccoon dog-associated TTV was identified for the first time in lung tissue.

## Data Availability

The datasets presented in this study can be found in online repositories. The names of the repository/repositories and accession number(s) can be found in the article/Supplementary material.

## Footnotes

^1^ https://github.com/shihuang047/2bRAD-M

^2^ https://nmdc.cn/gcpathogen/
