# Supplementary material for: Lung microbiota of raccoon dogs (Nyctereutes procyonoides) using high-throughput sequencing
Source: Front Microbiol. 2025 Oct 20;16:1677761. doi: 10.3389/fmicb.2025.1677761 (PMC12580213; doi:10.3389/fmicb.2025.1677761)
Supplement: Supplementary file 1 [file Table_1.DOCX]

Supplementary Material

# Supplementary Tables

**Appendix Table.**

Table S1 Raccoon dog lung tissue sampling log

| Group | Sample name | Source location | Collection time |
| --- | --- | --- | --- |
| 1 | R1F1 | Hou Ying Breeding Farm, Dongye Gully, Pingshan County | 2022.5.8 |
|  | R2F1 | Hou Ying Breeding Farm, Dongye Gully, Pingshan County | 2022.5.8 |
| 2 | R3F1 | Zhongshan Mink and Fox Farmers' Cooperative | 2019.12.1 |
|  | R4F1 | Zhongshan Mink and Fox Farmers' Cooperative | 2019.12.1 |
|  | R5F1 | Zhongshan Mink and Fox Farmers' Cooperative | 2019.12.1 |
|  | R6F1 | Zhongshan Mink and Fox Farmers' Cooperative | 2019.12.1 |
|  | R7F1 | Zhongshan Mink and Fox Farmers' Cooperative | 2019.12.1 |
|  | R8F1 | Zhongshan Mink and Fox Farmers' Cooperative | 2019.12.1 |
|  | R9F1 | Zhongshan Mink and Fox Farmers' Cooperative | 2019.12.1 |
| 3 | R10F1 | Li Xinchao Breeding Farm, Nandian Village, Pingshan County | 2021.06.29 |
| 4 | R11F1 | Qin Qiusheng Breeding Farm, Pingshan County | 2021.7.13 |
| 5 | R12F1 | Chenzhuang Raccoon Dog Breeding Farm, Lingshou County | 2020.6.16 |
| 6 | R13F1 | Hou Ying Breeding Farm, Dongye Gully, Pingshan County | 2020.6. 23 |
|  | R14F1 | Hou Ying Breeding Farm, Dongye Gully, Pingshan County | 2020.6.23 |
| 7 | R15F1 | Liu Wentao Breeding Farm, Pingshan Town, Pingshan County | 2023.8.10 |
| 8 | R16F1 | Zheng Dan Farm, Pingshan County | 2023.8.20 |
|  | R17F1 | Zheng Dan Farm, Pingshan County | 2023.8.20 |
|  | R18F1 | Zheng Dan Farm, Pingshan County | 2023.8.20 |
| 9 | R19F1 | Wang Yanping Breeding Farm, Xiadongyu Village, Pingshan County | 2023.8.31 |
|  | R20F1 | Wang Yanping Breeding Farm, Xiadongyu Village, Pingshan County | 2023.8.31 |
|  | R21F1 | Wang Yanping Breeding Farm, Xiadongyu Village, Pingshan County | 2023.8.31 |
|  | R22F1 | Wang Yanping Breeding Farm, Xiadongyu Village, Pingshan County | 2023.8.31 |
|  | R23F1 | Wang Yanping Breeding Farm, Xiadongyu Village, Pingshan County | 2023.8.31 |
|  | R24F1 | Wang Yanping Breeding Farm, Xiadongyu Village, Pingshan County | 2023.8.31 |
|  | R25F1 | Wang Yanping Breeding Farm, Xiadongyu Village, Pingshan County | 2023.8.31 |
|  | R26F1 | Wang Yanping Breeding Farm, Xiadongyu Village, Pingshan County | 2023.8.31 |
| 10 | R27F1 | Shi Yuncui Breeding Farm, Sujiazhuang Village, Pingshan County | 2023.9.10 |
|  | R28F1 | Shi Yuncui Breeding Farm, Sujiazhuang Village, Pingshan County | 2023.9.10 |
|  | R29F1 | Shi Yuncui Breeding Farm, Sujiazhuang Village, Pingshan County | 2023.9.10 |
|  | R30F1 | Shi Yuncui Breeding Farm, Sujiazhuang Village, Pingshan County | 2023.9.10 |

Table S2. Change in data volume during quality control: raw reads, enzyme reads, and clean reads and percentage of clean reads

| Sample | Raw reads | Enzyme reads | Clean reads | Percent |
| --- | --- | --- | --- | --- |
| R1F1 | 8528695 | 8056281 | 7729099 | 90.62% |
| R2F1 | 7808165 | 7283395 | 6993864 | 89.57% |
| R3F1 | 10231718 | 9532371 | 9154064 | 89.47% |
| R4F1 | 8819195 | 8404175 | 8057151 | 91.36% |
| R5F1 | 8868904 | 8363236 | 8030957 | 90.55% |
| R6F1 | 8627106 | 8221594 | 7884452 | 91.39% |
| R7F1 | 8028318 | 7485611 | 7180030 | 89.43% |
| R8F1 | 10347827 | 9834556 | 9429090 | 91.12% |
| R9F1 | 9899621 | 9445735 | 9066298 | 91.58% |
| R10F1 | 11592795 | 11026669 | 10595399 | 91.40% |
| R11F1 | 11398388 | 10631542 | 10207385 | 89.55% |
| R12F1 | 10129581 | 9977609 | 9566257 | 94.44% |
| R13F1 | 9689237 | 8934978 | 8582786 | 88.58% |
| R14F1 | 7014602 | 6792481 | 6514930 | 92.88% |
| R15F1 | 7521506 | 6949454 | 6650252 | 88.42% |
| R16F1 | 8097531 | 7747110 | 7406583 | 91.47% |
| R17F1 | 9038053 | 8786598 | 8404917 | 92.99% |
| R18F1 | 7875236 | 7581445 | 7249086 | 92.05% |
| R19F1 | 10661946 | 10419247 | 9982084 | 93.62% |
| R20F1 | 8956943 | 8669981 | 8315967 | 92.84% |
| R21F1 | 9109807 | 8474612 | 8115317 | 89.08% |
| R22F1 | 10861316 | 10532447 | 10023947 | 92.29% |
| R23F1 | 12840300 | 12110028 | 11522800 | 89.74% |
| R24F1 | 12598650 | 12375543 | 11774114 | 93.46% |
| R25F1 | 15089873 | 14823574 | 14107694 | 93.49% |
| R26F1 | 13125673 | 12343659 | 11746709 | 89.49% |
| R27F1 | 16327381 | 15999210 | 15255661 | 93.44% |
| R28F1 | 14700076 | 14064640 | 13380547 | 91.02% |
| R29F1 | 15493353 | 14889123 | 14194595 | 91.62% |
| R30F1 | 10869148 | 10640062 | 10122838 | 93.13% |
| Extraction blank | 159658 | 590 | 561 | 0.35% |
| Library blank | 96974 | 7128 | 6772 | 6.98% |

Table S3. Microbial composition of raccoon dog lungs between farms at phylum, genus and levels

| Name | | Relative abundance of group 2 | Relative abundance of group 8 | Relative  abundance of group 9 | Relative abundance of group 10 | P-value |
| --- | --- | --- | --- | --- | --- | --- |
| Phylum | Pseudomonadota | 0.63±0.16 | 0.84± 0.05 | 0.75±0.14 | 0.70±0.11 | 0.092 |
|  | Ascomycota | 0.12±0.05 | 0.07±0.05 | 0.13±0.05 | 0.16±0.08 | 0.308 |
|  | Actinomycetota | 0.09±0.06 | 0.01±0.01 | 0.05±0.09 | 0.09±0.09 | 0.116 |
|  | Bacillota | 0.07±0.13 | 0.07±0.11 | 0.02±0.03 | 0.02±0.02 | 0.509 |
|  | Chlamydiota | 0.05±0.10 | 0 | 0 | 0 | 0.213 |
|  | Bacteroidota | 0.03^a^±0.01 | 0.01 ±0.02 | 0.01^b^±0.01 | 0.02 ± 0.08 | 0.003 |
| Genus | Acinetobacter | 0.36±0.18 | 0.35±0.32 | 0.42±0.15 | 0.25±0.20 | 0.577 |
|  | Escherichia | 0.09±0.10 | 0.26±0.24 | 0.11±0.08 | 0.03±0.01 | 0.091 |
|  | Klebsiella | 0.05±0.05 | 0.11±0.12 | 0.07±0.05 | 0.21±0.24 | 0.454 |
|  | Pichia | 0.10±0.05 | 0.06±0.04 | 0.11±0.04 | 0.13±0.07 | 0.688 |
|  | Ralstonia | 0.05±0.03 | 0.06±0.07 | 0.04±0.06 | 0.07±0.10 | 0.075 |
|  | Rothia | 0.07 ^a^±0.04 | 0.01±0.01 | 0.03 ^b^±0.08 | 0.05±0.06 | 0.034 |
|  | Bradyrhizobium | 0.02 ±0.01 | 0.02±0.01 | 0.06±0.04 | 0.06±0.03 | 0.075 |
|  | Chlamydophila | 0.05±0.10 | 0 | 0 | 0 | 0.194 |
|  | Streptococcus | 0.04±0.06 | 0 | 0.01±0.03 | 0±0.01 | 0.07 |
|  | Talaromyces | 0.01±0 | 0.01±0.01 | 0.01±0.01 | 0.02±0.01 | 0.382 |
| Species | Acinetobacter baumannii | 0.36±0.18 | 0.35±0.31 | 0.42±0.15 | 0.25±0.20 | 0.526 |
|  | Escherichia coli | 0.09 ± 0.1 | 0.26±0.24 | 0.11±0.08 | 0.03 ±0.01 | 0.034 |
|  | Klebsiella pneumoniae | 0.05±0.05 | 0.07±0.06 | 0.07±0.05 | 0.21±0.24 | 0.312 |
|  | Pichia inconspicua | 0.10±0.05 | 0.06±0.04 | 0.11±0.04 | 0.13±0.07 | 0.357 |
|  | Ralstonia sp000620465 | 0.03±0.02 | 0.04±0.04 | 0.03±0.04 | 0.05±0.06 | 0.576 |
|  | Bradyrhizobium sp003020075 | 0.02 ±0.01 | 0.02±0.02 | 0.05±0.03 | 0.04±0.02 | 0.123 |
|  | Chlamydophila abortus | 0.05±0.10 | 0 | 0 | 0 | 0.213 |
|  | Rothia sp902373285 | 0.06 ^a^±0.03 | 0.01±0.01 | 0 ^b^ | 0 ^b^ | 0;0.006 |
|  | Talaromyces rugulosus | 0.01±0 | 0.01±0.01 | 0.01±0.01 | 0.02±0.01 | 0.382 |
|  | Fusobacterium A sp900015295 | 0 | 0 | 0.04±0.13 | 0 | 0.626 |
|  | Rothia dentocariosa | 0.01± 0.01 | 0 | 0.02±0.04 | 0.04± 00.04 | 0.057 |
|  | Pelomonas sp003963075 | 0.01± 0.02 | 0 | 0 | 0.01±0.01 | 0.081 |
|  | Fusarium oxysporum | 0.01±0 | 0.01±0 | 0.01±0 | 0.01±0 | 0.305 |
|  | Afipia broomeae | 0 | 0 | 0.01±0.01 | 0.02±0.02 | 0.052 |
|  | Ralstonia pickettii | 0.01±0.01 | 0.01±0.01 | 0.01±0.01 | 0.01±0.02 | 0.855 |
|  | Streptococcus lactarius | 0.02 ^a^±0.02 | 0 | 0 ^b^ | 0 | 0.033 |
|  | Rothia aeria | 0 | 0 | 0.01±0.04 | 0.01±0.02 | 0.155 |
|  | Salmonella enterica | 0.02±0.05 | 0 | 0±0.05 | 0±0.01 | 0.863 |
|  | Alloprevotella sp905371275 | 0.01 ^a^±0.01 | 0.01±0.01 | 0 ^b^ | 0 ^b^ | 0.005;0.035 |
|  | Prevotella histicola | 0.01 ^a^±0.01 | 0 | 0 ^b^ | 0 | 0.033 |
|  | Klebsiella quasipneumoniae | 0 | 0.04±0.08 | 0 | 0 | 0.096 |
|  | Ralstonia mannitolilytica | 0 | 0±0.01 | 0 | 0.01±0.01 | 0.656 |
|  | Bradyrhizobium sp016462955 | 0 | 0 | 0.01±0.01 | 0.01±0.01 | 0.043 |
|  | Lactococcus petauri | 0 | 0.04±0.06 | 0 | 0 | 0.096 |
|  | Lautropia mirabilis | 0 ^a^ | 0 | 0.01±0.02 | 0.01^b^±0.02 | 0.024 |
|  | Sediminibacterium sp017537025 | 0 | 0.01±0.01 | 0±0.01 | 0.01±0.01 | 0.036 |
|  | Phyllobacterium calauticae | 0 ^a^ | 0 | 0.01 ^b^±0.01 | 0.01±0.01 | 0.008 |
|  | Nocardioides sp000519005 | 0 | 0±0.01 | 0.01±0.01 | 0.01±0.01 | 0.734 |
|  | Stenotrophomonas pavanii | 0 | 0 | 0±0.01 | 0.02±0.03 | 0.157 |

Table S4. The summary of library information of raccoon dogs

| Library ID | Sample type | No.of sample | Healthy status | Total no. of raw reads | Filtering reads | Clean reads |
| --- | --- | --- | --- | --- | --- | --- |
| 1 | Lung | 10 | Sick | 58512218 | 56042140 | 28021070 |
| 2 | Lung | 10 | Sick | 75895168 | 69573170 | 34786585 |
| 3 | Lung | 10 | Sick | 65659632 | 61543548 | 30771774 |
