# Supplementary material for: Lung microbiota of raccoon dogs (Nyctereutes procyonoides) using high-throughput sequencing
Source: Front Microbiol. 2025 Oct 20;16:1677761. doi: 10.3389/fmicb.2025.1677761 (PMC12580213; doi:10.3389/fmicb.2025.1677761)
Supplement: Supplementary file 2 [file Data_Sheet_1.pdf]

Supplementary Material

Supplementary Tables

Appendix Table.

Table S1 Raccoon dog lung tissue sampling log

Group	Sample name	Source location	Collection time
1	R1F1	Hou Ying Breeding Farm, Dongye Gully, Pingshan County	2022.5.8
	R2F1	Hou Ying Breeding Farm, Dongye Gully, Pingshan County	2022.5.8
2	R3F1	Zhongshan Mink and Fox Farmers' Cooperative	2019.12.1
	R4F1	Zhongshan Mink and Fox Farmers' Cooperative	2019.12.1
	R5F1	Zhongshan Mink and Fox Farmers' Cooperative	2019.12.1
	R6F1	Zhongshan Mink and Fox Farmers' Cooperative	2019.12.1
	R7F1	Zhongshan Mink and Fox Farmers' Cooperative	2019.12.1
	R8F1	Zhongshan Mink and Fox Farmers' Cooperative	2019.12.1
	R9F1	Zhongshan Mink and Fox Farmers' Cooperative	2019.12.1
3	R10F1	Li Xinchao Breeding Farm, Nandian Village, Pingshan County	2021.06.29
4	R11F1	Qin Qiusheng Breeding Farm, Pingshan County	2021.7.13
5	R12F1	Chenzhuang Raccoon Dog Breeding Farm, Lingshou County	2020.6.16
6	R13F1	Hou Ying Breeding Farm, Dongye Gully, Pingshan County	2020.6. 23
	R14F1	Hou Ying Breeding Farm, Dongye Gully, Pingshan County	2020.6.23
7	R15F1	Liu Wentao Breeding Farm, Pingshan Town, Pingshan County	2023.8.10
8	R16F1	Zheng Dan Farm, Pingshan County	2023.8.20
	R17F1	Zheng Dan Farm, Pingshan County	2023.8.20

	R18F1	Zheng Dan Farm, Pingshan County	2023.8.20
9	R19F1	Wang Yanping Breeding Farm, Xiadongyu Village, Pingshan County	2023.8.31
	R20F1	Wang Yanping Breeding Farm, Xiadongyu Village, Pingshan County	2023.8.31
	R21F1	Wang Yanping Breeding Farm, Xiadongyu Village, Pingshan County	2023.8.31
	R22F1	Wang Yanping Breeding Farm, Xiadongyu Village, Pingshan County	2023.8.31
	R23F1	Wang Yanping Breeding Farm, Xiadongyu Village, Pingshan County	2023.8.31
	R24F1	Wang Yanping Breeding Farm, Xiadongyu Village, Pingshan County	2023.8.31
	R25F1	Wang Yanping Breeding Farm, Xiadongyu Village, Pingshan County	2023.8.31
	R26F1	Wang Yanping Breeding Farm, Xiadongyu Village, Pingshan County	2023.8.31
10	R27F1	Shi Yuncui Breeding Farm, Sujiazhuang Village, Pingshan County	2023.9.10
	R28F1	Shi Yuncui Breeding Farm, Sujiazhuang Village, Pingshan County	2023.9.10
	R29F1	Shi Yuncui Breeding Farm, Sujiazhuang Village, Pingshan County	2023.9.10
	R30F1	Shi Yuncui Breeding Farm, Sujiazhuang Village, Pingshan County	2023.9.10

Table S2. Change in data volume during quality control: raw reads, enzyme reads, and clean reads and percentage of clean reads

Sample	Raw reads	Enzyme reads	Clean reads	Percent
R1F1	8528695	8056281	7729099	90.62%
R2F1	7808165	7283395	6993864	89.57%
R3F1	10231718	9532371	9154064	89.47%
R4F1	8819195	8404175	8057151	91.36%
R5F1	8868904	8363236	8030957	90.55%

R6F1	8627106	8221594	7884452	91.39%
R7F1	8028318	7485611	7180030	89.43%
R8F1	10347827	9834556	9429090	91.12%
R9F1	9899621	9445735	9066298	91.58%
R10F1	11592795	11026669	10595399	91.40%
R11F1	11398388	10631542	10207385	89.55%
R12F1	10129581	9977609	9566257	94.44%
R13F1	9689237	8934978	8582786	88.58%
R14F1	7014602	6792481	6514930	92.88%
R15F1	7521506	6949454	6650252	88.42%
R16F1	8097531	7747110	7406583	91.47%
R17F1	9038053	8786598	8404917	92.99%
R18F1	7875236	7581445	7249086	92.05%
R19F1	10661946	10419247	9982084	93.62%
R20F1	8956943	8669981	8315967	92.84%
R21F1	9109807	8474612	8115317	89.08%
R22F1	10861316	10532447	10023947	92.29%
R23F1	12840300	12110028	11522800	89.74%
R24F1	12598650	12375543	11774114	93.46%
R25F1	15089873	14823574	14107694	93.49%
R26F1	13125673	12343659	11746709	89.49%
R27F1	16327381	15999210	15255661	93.44%

R28F1	14700076	14064640	13380547	91.02%
R29F1	15493353	14889123	14194595	91.62%
R30F1	10869148	10640062	10122838	93.13%
Extraction blank	159658	590	561	0.35%
Library blank	96974	7128	6772	6.98%

Table S3. Microbial composition of raccoon dog lungs between farms at phylum, genus and levels

	Name	Relative abundance of group 2	Relative abundance of group 8	Relative abundance of group 9	Relative abundance of group 10	P-value
Phylum	Pseudomonadota	0.63±0.16	0.84± 0.05	0.75±0.14	0.70±0.11	0.092
	Ascomycota	0.12±0.05	0.07±0.05	0.13±0.05	0.16±0.08	0.308
	Actinomycetota	0.09±0.06	0.01±0.01	0.05±0.09	0.09±0.09	0.116
	Bacillota	0.07±0.13	0.07±0.11	0.02±0.03	0.02±0.02	0.509
	Chlamydiota	0.05±0.10	0	0	0	0.213
	Bacteroidota	0.03^{a}±0.01	0.01 ±0.02	0.01^{b}±0.01	0.02 ± 0.08	0.003
Genus	Acinetobacter	0.36±0.18	0.35±0.32	0.42±0.15	0.25±0.20	0.577
	Escherichia	0.09±0.10	0.26±0.24	0.11±0.08	0.03±0.01	0.091
	Klebsiella	0.05±0.05	0.11±0.12	0.07±0.05	0.21±0.24	0.454
	Pichia	0.10±0.05	0.06±0.04	0.11±0.04	0.13±0.07	0.688
	Ralstonia	0.05±0.03	0.06±0.07	0.04±0.06	0.07±0.10	0.075
	Rothia	0.07 ^{a}±0.04	0.01±0.01	0.03 ^{b}±0.08	0.05±0.06	0.034
	Bradyrhizobium	0.02 ±0.01	0.02±0.01	0.06±0.04	0.06±0.03	0.075
	Chlamydophila	0.05±0.10	0	0	0	0.194
	Streptococcus	0.04±0.06	0	0.01±0.03	0±0.01	0.07
	Talaromyces	0.01±0	0.01±0.01	0.01±0.01	0.02±0.01	0.382
Species	Acinetobacter baumannii	0.36±0.18	0.35±0.31	0.42±0.15	0.25±0.20	0.526
	Escherichia coli	0.09 ± 0.1	0.26±0.24	0.11±0.08	0.03 ±0.01	0.034

Klebsiella pneumoniae	0.05±0.05	0.07±0.06	0.07±0.05	0.21±0.24	0.312
Pichia inconspicua	0.10±0.05	0.06±0.04	0.11±0.04	0.13±0.07	0.357
Ralstonia sp000620465	0.03±0.02	0.04±0.04	0.03±0.04	0.05±0.06	0.576
Bradyrhizobium sp003020075	0.02 ±0.01	0.02±0.02	0.05±0.03	0.04±0.02	0.123
Chlamydophila abortus	0.05±0.10	0	0	0	0.213
Rothia sp902373285	0.06 [a]±0.03	0.01±0.01	0 [b]	0 [b]	0;0.006
Talaromyces rugulosus	0.01±0	0.01±0.01	0.01±0.01	0.02±0.01	0.382
Fusobacterium A sp900015295	0	0	0.04±0.13	0	0.626
Rothia dentocariosa	0.01± 0.01	0	0.02±0.04	0.04± 00.04	0.057
Pelomonas sp003963075	0.01± 0.02	0	0	0.01±0.01	0.081
Fusarium oxysporum	0.01±0	0.01±0	0.01±0	0.01±0	0.305
Afipia broomeae	0	0	0.01±0.01	0.02±0.02	0.052
Ralstonia pickettii	0.01±0.01	0.01±0.01	0.01±0.01	0.01±0.02	0.855
Streptococcus lactarius	0.02 [a]±0.02	0	0 [b]	0	0.033
Rothia aeria	0	0	0.01±0.04	0.01±0.02	0.155
Salmonella enterica	0.02±0.05	0	0±0.05	0±0.01	0.863
Alloprevotella sp905371275	0.01 [a]±0.01	0.01±0.01	0 [b]	0 [b]	0.005; 0.035
Prevotella histicola	0.01 [a]±0.01	0	0 [b]	0	0.033
Klebsiella quasipneumoniae	0	0.04±0.08	0	0	0.096
Ralstonia mannitolilytica	0	0±0.01	0	0.01±0.01	0.656
Bradyrhizobium sp016462955	0	0	0.01±0.01	0.01±0.01	0.043
Lactococcus petauri	0	0.04±0.06	0	0	0.096
Lautropia mirabilis	0 [a]	0	0.01±0.02	0.01[b]±0.02	0.024
Sediminibacterium sp017537025	0	0.01±0.01	0±0.01	0.01±0.01	0.036
Phyllobacterium calauticae	0 [a]	0	0.01 [b]±0.01	0.01±0.01	0.008
Nocardioides sp000519005	0	0±0.01	0.01±0.01	0.01±0.01	0.734
Stenotrophomonas pavanii	0	0	0±0.01	0.02±0.03	0.157

Table S4. The summary of library information of raccoon dogs

Library ID	Sample type	No.of sample	Healthy status	Total no. of raw reads	Filtering reads	Clean reads

1	Lung	10	Sick	58512218	56042140	28021070
2	Lung	10	Sick	75895168	69573170	34786585
3	Lung	10	Sick	65659632	61543548	30771774
